# Influence of socioeconomic factors on medically unnecessary ambulance calls

**DOI:** 10.1186/1472-6963-7-120

**Published:** 2007-07-27

**Authors:** Chihiro Kawakami, Kenji Ohshige, Katsuaki Kubota, Osamu Tochikubo

**Affiliations:** 1 Department of Public Health, Yokohama City University School of Medicine, 3–9 Fukuura, Kanazawa-ku, Yokohama 236-0004, Japan; 2 Research Group of Ambulance Service, National Research Institute of Fire and Disaster, 3-14-1 Nakahara, Mitaka-city, Tokyo 181-8633, Japan

## Abstract

**Background:**

Unnecessary ambulance use has become a socioeconomic problem in Japan. We investigated the possible relations between socioeconomic factors and medically unnecessary ambulance calls, and we estimated the incremental demand for unnecessary ambulance use produced by socioeconomic factors.

**Methods:**

We conducted a self-administered questionnaire-based survey targeting residents of Yokohama, Japan. The questionnaire included questions pertaining to socioeconomic characteristics, dichotomous choice method questions pertaining to ambulance calls in hypothetical nonemergency situations, and questions on the city's emergency medical system. The probit model was used to analyze the data.

**Results:**

A total of 2,029 out of 3,363 targeted recipients completed the questionnaire (response rate, 60.3%). Probit regression analyses showed that several demographic and socioeconomic factors influence the decision to call an ambulance. Male respondents were more apt than female respondents to state that they would call an ambulance in nonemergency situations (p < 0.05). Age was an important factor influencing the hypothetical decision to call an ambulance (p < 0.05); elderly persons were more apt than younger persons to state that they would call an ambulance. Possession of a car and hesitation to use an ambulance negatively influenced the hypothetical decision to call an ambulance (p < 0.05). Persons who do not have a car were more likely than those with a car to state that they would call an ambulance in unnecessary situations.

**Conclusion:**

Results of the study suggest that several socioeconomic factors, i.e., age, gender, household income, and possession of a car, influence a person's decision to call an ambulance in nonemergency situations. Hesitation to use an ambulance and knowledge of the city's primary emergency medical center are likely to be important factors limiting ambulance overuse. It was estimated that unnecessary ambulance use is increased approximately 10% to 20% by socioeconomic factors.

## Background

Increased demand for emergency medical services has become a common problem in industrialized countries [1-14]. Growing demand for prehospital emergency medical services has also been observed. Fischer et al. [10] reported that demand for ambulances increased at a rate of about 4% each year throughout most of the last decade in the United Kingdom. Clark et al. [3] documented an increase in the cost of ambulance services in Australia from 393 million to 523 million US dollars over a 5-year period.

In Yokohama, Japan, the number of ambulances dispatched is rapidly increasing, and the increase has become a socioeconomic problem [15]. Although the main reason for the growing demand for ambulances is growth of the elderly population [15], unnecessary ambulance calls also increase the number of ambulances dispatched. There were 157,371 calls to the ambulance service of the Yokohama Fire Bureau in 2004 [16], and according to the Bureau's emergency medical transport records, approximately 60% of transported individuals were able to return home without special treatment or hospitalization [17]. The Bureau considers most of these cases to have been nonurgent, i.e., to represent unnecessary ambulance use.

The problem of inappropriate ambulance use has been reported worldwide [18-24]. Brown et al. [23] reported that inappropriate ambulance use accounts for 40% to 50% of total ambulance use in the United States, Canada, Sweden, and England. Several studies have indicated that ambulance misuse or overuse is related to socioeconomic characteristics of the users. Billittier et al. [20] reported that individuals in the United States who are covered by medical assistance (Medicaid) tend to use ambulances unnecessarily. Camasso-Richardson et al. [24] reported that individuals who do not have a private car are apt to use ambulances unnecessarily.

The aims of this study were to determine what factors account for a person's decision to call an ambulance in situations in which the call is actually inappropriate and to estimate the incremental demand for unnecessary ambulance use produced by socioeconomic factors.

## Methods

### Emergency ambulance system in Japan

In Japan, local governments organize emergency ambulance service as a public service. Anyone can use an ambulance free of charge by making a phone call to "119". Ambulance crews are dispatched for all emergency calls, and all patients attended by a crew are transported to a hospital unless they refuse. Most local governments staff ambulances with emergency life-saving technicians who are trained for cardiopulmonary resuscitation [15,25,26].

### Study site and study design

The study was conducted in Yokohama, Japan, which has a population of 3,579,133 (2005 census) and covers an area of 434 square kilometers. In Yokohama, the number of ambulances dispatched in 2005 was 1.58 times greater than that in 1995 [16], although the population in 2005 was only 1.08 times greater than that in 1995 [27]. The city's ambulance transport service is unified and managed by the Emergency Medical Division of the Yokohama Fire Bureau.

We conducted an anonymous self-administered questionnaire-based survey targeting 3,600 city residents randomly selected from the city resident registration list; 1,200 city residents were extracted from each of three age groups, 20–39 years, 40–64 years, and 65 years or over. Questionnaires were distributed once and returned in September 2004. Reminders were not sent. Detailed information about sampling for the study has been published elsewhere [28].

### Questionnaire

The questionnaire consisted of three parts. The first part included questions on demographic and socioeconomic characteristics such as the respondent's age, gender, and pretax annual household income.

The second part included hypothetical questions pertaining to ambulance use. We applied the dichotomous choice method to estimate the probability of calling an ambulance [29-31]. Three hypothetical nonemergency situations were presented. Scenario 1 represented a situation in which the respondent faces a nonserious condition such as an ankle sprain or bruise on the leg. Scenario 2 represented a situation in which the respondent's child or young relative faces a nonserious condition such as a nose cold. Scenario 3 represented a situation in which the respondent's elderly relative faces a nonserious situation such as lack of transport to a clinic. The influence of user charges was assessed along with the hypothetical questions by presentation of one of eight possible prices ($0, $9.50, $28.50, $47.50, $95.00, $190.00, $285.00, and $475.00; $1 = ¥105) for use of an ambulance. Each respondent was shown only one price randomly selected from the eight prices for each of the three hypothetical scenarios. For each situation, respondents answered "yes" if they would call an ambulance or "no" if they would not. The three hypothetical scenarios were as follows:

Scenario 1: At around 6 a.m., you miss your step and tumble down the stairs. You then develop pain in your legs, but you can walk with difficulty. If you were in this situation, would you call an ambulance?

Scenario 2: One morning, a child who is living with you develops a cough and nasal discharge. You give him/her a drug, which has been kept at home for future use, but the symptoms do not disappear even by 8 p.m. on the same day. If you were in this situation, would you call an ambulance? Even if you do not live with a child, please answer the question as if you do.

Scenario 3: It's around 8 a.m. An elderly person who is living with you needs to go to the hospital to see his/her primary care physician. Normally, someone in your family would drive the elderly person to the hospital, but no one can do so today. If you were in this situation, would you call an ambulance? Even if you do not live with an elderly person, please answer the question as if you do.

The third part included questions on the city's emergency medical system, such as "Have you ever used an ambulance?" "Do you feel hesitant to call an ambulance?" "Are you familiar with the primary emergency medical centers?" "Are you familiar with the emergency medical information center?" and "Do you know of any private patient transport service?"

### Analysis of the influence of socioeconomic factors

The probability P of a "yes" response to the dichotomous choice questions was expressed by a probit model [32,33]:

P(Y = 1) = Φ(*β*_0 _+ *β*_1_Price + *β*_2_Gender + *β*_3_Age + *β*_4_Family + *β*_5_Income + *β*_6_Car + *β*_7_History + *β*_8_Hesitation + *β*_9_E.Center + *β*_10_E.Info + *β*_11_P.Trans)

where "Y" equals 1 if the respondent chose "yes" and 0 if otherwise. Φ is the standard cumulative normal distribution, and β_n _is the estimated parameter vector. Price is the hypothetical price of ambulance use and is treated as a quantitative variable. Gender is a dummy variable (male = 1, female = 0). Age is the age group that the respondent belongs to, with 1 representing 20–29 years, 2 representing 30–39 years, 3 representing 40–49 years, 4 representing 50–59 years, 5 representing 60–69 years, 6 representing 70–79 years, and 7 representing 80 years and over. Family is a dummy variable for family structure: living alone was expressed as 1 (other = 0), when the response to Scenario 1 was analyzed; living with children aged 5 years or younger was expressed as 1 (other = 0) when the response to Scenario 2 was analyzed; living with a relative aged 65 years or older was expressed as 1 (other = 0) when the response to Scenario 3 was analyzed. Income is the pretax annual household income, expressed as 1 if less than $19,000, 2 if $19,000 to $37,999, 3 if $38,000 to $56,999, 4 if $57,000 to $75,999, 5 if $76,000 to $94,999, and 6 if $95,000 or more. Car is a dummy variable for possession of a car by which the respondent could visit a hospital or clinic (yes = 1, no = 0). History is a dummy variable for the respondent's past ambulance use (yes = 1, no = 0). Hesitation is a dummy variable for feeling hesitant to use an ambulance (yes = 1, no = 0). E.Center is a dummy variable for knowledge of a primary emergency medical center (yes = 1, no = 0). E.Info is a dummy variable for knowledge of the emergency medical information center, which serves as a directory where individuals can obtain the name of a suitable hospital or clinic (yes = 1, no = 0). P.Trans is a dummy variable for knowledge of a private patient transport service (yes = 1, no = 0).

The marginal effect of each factor, that is, a change in the probability of a "yes" response (for an independent binary variable) or of a one-unit increase (for an independent ordinal variable), i.e. estimated percentage of incremental demands for ambulance use produced by socioeconomic factors, was also computed with the probit model.

### Statistical analysis

STATA/SE 8.2 software for Windows (Stata Corp., College Station, Texas, USA) was used for all statistical analyses including probit regression analysis. P values of less than 0.05 were considered statistically significant.

### Ethics

The questionnaire-based study was conducted anonymously. No personal information, such as the respondent's name or address, was contained in the returned questionnaires. Analysis of ambulance calls to the Yokohama Fire Bureau to estimate incremental demand was also conducted anonymously. The study was approved by the Committee on the Regional Emergency Medical Service System of Yokohama after ethical aspects of the study were reviewed by the Human Rights Affairs Division, Civic Affairs Bureau, City of Yokohama.

## Results

### Characteristics of respondents

Questionnaires were distributed to 3,600 residents, 237 questionnaires were undeliverable because of incorrect addresses. Of the 3,363 questionnaires, 2,029 were completed and returned (response rate, 60.3%). Respondent characteristics are summarized in Table 1. Two hundred thirty-one respondents (11.4%) were living with at least one child aged 5 years or younger, and 795 respondents (39.2%) were living with at least one relative aged 65 years or older. A total of 1,279 respondents (63.0%) owned a car.

**Table 1:** Demographic and socioeconomic characteristics of survey respondents.

	Number	(%)
Gender		
Male	911	(44.9%)
Female	1,112	(54.8%)
No answer	6	(0.3%)
Age (years)		
20 to 29	168	(8.3%)
30 to 39	315	(15.5%)
40 to 49	239	(11.8%)
50 to 59	321	(15.8%)
60 to 69	378	(18.6%)
70 to 79	439	(21.6%)
80 or over	164	(8.1%)
No answer	5	(0.2%)
Living alone		
Yes	188	(9.3%)
No	1,824	(89.9%)
No answer	17	(0.8%)
Living with child aged 5 years or younger		
Yes	231	(11.4%)
No	1,721	(84.8%)
No answer	77	(3.8%)
Living with elderly person aged 65 years or older		
Yes	795	(39.2%)
No	1,155	(56.9%)
No answer	79	(3.9%)
Pretax annual household income (US $)		
Less than 19,000	163	(8.0%)
19,000 – 37,999	490	(24.1%)
38,000 – 56,999	395	(19.5%)
57,000 – 75,999	307	(15.1%)
76,000 – 94,999	208	(10.3%)
95,000 or more	301	(14.8%)
No answer	165	(8.1%)
Possession of a car		
Yes	1,279	(63.0%)
No	726	(35.8%)
No answer	24	(1.2%)
History of ambulance use		
Yes	949	(46.8%)
No	1,065	(52.5%)
No answer	15	(0.7%)
Hesitation to call an ambulance		
Yes	953	(47.0%)
No	1,051	(51.8%)
No answer	25	(1.2%)
Knowledge of primary emergency medical center		
Yes	1,163	(57.3%)
No	837	(41.3%)
No answer	29	(1.4%)
Knowledge of emergency medical information center		
Yes	467	(23.0%)
No	1,534	(75.6%)
No answer	28	(1.4%)
Knowledge of private medical transport service		
Yes	167	(8.2%)
No	1,847	(91.0%)
No answer	15	(0.7%)

### Number of respondents who stated they would have called an ambulance

A "yes" response (i.e., that the individual would call an ambulance) was obtained from 240 respondents (11.8% of the total respondents) for Scenario 1, 255 respondents (12.6% of the total respondents) for Scenario 2, and 124 respondents (6.1% of the total respondents) for Scenario 3. The proportion who stated they would have called an ambulance was likely to decrease beyond the hypothetical price of $95, although a continuous downward effect of price on the decision to call an ambulance was not shown (Table 2).

**Table 2:** Number of respondents who stated they would have called an ambulance for each of the three scenarios per hypothetical price.

Hypothetical price ($)	Number of respondents	Call for Senario 1 Number (%)		Call for Senario 2 Number (%)		Call for Senario 3 Number (%)	
0.00	248	35	(14.1%)	38	(15.3%)	17	(6.9%)
9.50	252	34	(13.5%)	26	(10.3%)	14	(5.6%)
28.50	259	34	(13.1%)	39	(15.1%)	18	(6.9%)
47.50	237	29	(12.2%)	37	(15.6%)	20	(8.4%)
95.00	250	38	(15.2%)	30	(12.0%)	16	(6.4%)
190.00	253	25	(9.9%)	31	(12.3%)	14	(5.5%)
285.00	258	26	(10.%1)	28	(10.9%)	12	(4.7%)
475.00	272	19	(7.0%)	26	(9.6%)	13	(4.8%)
Total	2,029	240	(11.8%)	255	(12.6%)	124	(6.1%)

### Factors influencing unnecessary ambulance calls

The results of probit regression modeling for estimating the impact of factors likely to influence the decision to call an ambulance are shown in Table 3. The marginal effect of price was 0.00 for Scenarios 1, 2, and 3; thus, a downward effect of price was not observed.

**Table 3:** Influence of socioeconomic factors on the decision to call an ambulance in unnecessary situations.

Scenario 1	Marginal effect	95% CI		P-value
Price	0.000	0.000	0.000	0.026^#^
Gender	0.024	0.000	0.048	0.046
Age	0.039	0.031	0.046	0.000
Family (living alone)	0.055	0.002	0.107	0.015
Household income	0.002	-0.006	0.011	0.614
Possess a car	-0.044	-0.075	-0.014	0.002
History of ambulance use	0.015	-0.009	0.038	0.216
Hesitation to call an ambulance	-0.054	-0.078	-0.031	0.000
Knowledge of primary emergency medical center open at night	-0.012	-0.038	0.014	0.360
Knowledge of emergency medical information center	0.001	-0.030	0.032	0.952
Knowledge of private patient transport service	0.033	-0.017	0.083	0.148
Log likelihood	-512			
Pseudo R^2^	0.174			
Chi-square	215.6			
	(P < 0.001)			

Scenario 2	Marginal effect	95% CI		P-value

Price	0.000	0.000	0.000	0.132
Gender	0.047	0.020	0.074	0.001
Age	0.025	0.017	0.033	0.000
Family (living with at least one child)	0.009	-0.018	0.037	0.504
Household income	-0.016	-0.026	-0.007	0.001
Possess a car	-0.071	-0.105	-0.037	0.000
History of ambulance use	0.039	0.012	0.065	0.004
Hesitation to call an ambulance	-0.030	-0.056	-0.004	0.027
Knowledge of primary emergency medical center open at night	-0.042	-0.072	-0.012	0.005
Knowledge of emergency medical information center	0.001	-0.035	0.036	0.972
Knowledge of private patient transport service	0.015	-0.036	0.066	0.550
Log likelihood	-520			
Pseudo R^2^	0.148			
Chi-square	180.3			
	(P < 0.001)			

Scenario 3	Marginal effect	95% CI		P-value

Price	0.000	0.000	0.000	0.326
Gender	0.030	0.013	0.048	0.001
Age	0.013	0.007	0.018	0.000
Family (living with at least one elderly person)	0.013	-0.026	0.051	0.484
Household income	-0.006	-0.011	0.000	0.060
Possess a car	-0.023	-0.045	-0.002	0.018
History of ambulance use	-0.001	-0.018	0.015	0.860
Hesitation to call an ambulance	-0.024	-0.041	-0.007	0.006
Knowledge of primary emergency medical center open at night	-0.024	-0.045	-0.004	0.013
Knowledge of emergency medical information center	0.013	-0.013	0.040	0.276
Knowledge of private patient transport service	0.014	-0.021	0.050	0.374
Log likelihood	-313			
Pseudo R^2^	0.120			
Chi-square	85.7			
	(P < 0.001)			

CI: confidence interval.

# The marginal effect of price (= -8.16 × 10^-7^, standard error; 3.65 × 10^-7^, z-score; -2.22) was very small but statistically significant in Scenario 1.

In Scenario 1, i.e., when facing a nonserious situation, male respondents were more likely than female respondents to state that they would call an ambulance (p = 0.046); age of the respondent positively influenced the hypothetical decision to call an ambulance (p < 0.001); and respondents who lived alone were also more apt to state that they would call an ambulance (p = 0.015). Possession of a car and hesitation to call an ambulance had significant negative effects in Scenario 1 (p = 0.002 and p < 0.001, respectively). In Scenario 2, i.e., when a child or young relative faced a nonserious situation, male respondents were more likely than female respondents to state that they would call an ambulance (p = 0.001); age of the respondent and a history of ambulance use positively influenced the hypothetical decision to call an ambulance (p < 0.001 and p = 0.004, respectively). Household income, possession of a car, hesitation to call an ambulance, and knowledge of a primary emergency medical center had significant negative effects in Scenario 2 (p = 0.001, p < 0.001, p = 0.027, and p = 0.005, respectively). In Scenario 3, i.e., when an elderly relative faced a nonserious situation, male respondents were more likely than female respondents to state that they would call an ambulance (p = 0.001), and age of the respondent positively influenced the hypothetical decision to call an ambulance (p < 0.001). Possession of a car, hesitation to call an ambulance, and knowledge of a primary emergency medical center had significant negative effects in Scenario 3 (p = 0.018, p = 0.006, and p = 0.013, respectively). There was no evidence of multicolinearity; the sample correlation coefficient between any pair of variables was < +0.70 and > -0.70.

### Potential incremental demands for unnecessary ambulance use produced by socioeconomic factors

Marginal effects estimated by the probit model show the potential incremental demands produced by socioeconomic factors (Table 3). Analysis of responses to Scenario 1 indicated that the incremental demands due to age, living alone, nonpossession of a car, and nonhesitation to call were an estimated 3.9%, 5.5%, 4.4%, and 5.4%, respectively, of the unnecessary calls that individuals would have placed for themselves. These four factors together potentially produce 19.2% of the overall incremental demand. From the analysis of responses to Scenario 2, the incremental demands due to age, nonpossession of a car, nonhesitation to call, and lack of knowledge of primary emergency medical centers were estimated at 2.5%, 7.1%, 3.0%, and 4.2%, respectively, of the calls that family members would have made for children in unnecessary situations. The potential incremental demand produced by the four factors together was estimated at 16.8%. Similarly, the incremental demands due to age, nonpossession of a car, nonhesitation to call, and lack of knowledge of primary emergency medical centers estimated from the analysis of responses to Scenario 3 were 1.3%, 2.3%, 2.4%, and 2.4%, respectively, of the unnecessary calls that family members would have made for elderly relatives. The potential incremental demand produced by the four socioeconomic factors together was estimated at 8.4%.

## Discussion

It has been reported that unnecessary ambulance use is related to socioeconomic factors [18-24]. However, there have been few studies of how socioeconomic factors influence a person's decision to call an ambulance [28]. We analyzed the influence of various socioeconomic factors on decisions to call an ambulance in hypothetical nonemergency situations.

Age is an important factor influencing the demand for emergency medical services. The present study showed that elderly persons would tend to call an ambulance more easily than younger persons; age influenced the hypothetical ambulance call rate linearly. Victor et al. [5] reported that calls made by individuals aged 60 years or older accounted for 40% of all ambulance calls in London (England). McConnel et al. [14] reported that the emergency medical incident rate among persons aged 85 years or older was 3.4 times higher than that among persons aged 45 to 64 years in Texas (United States). Elderly persons naturally need more medical services, including emergency medical services, than younger persons need. They are also more likely to be risk sensitive and to call an ambulance in emergency situations [28]. The present study showed that age influences the decision to call an ambulance even in nonserious situations; the reason for this, however, remains unclear.

Economic status is another factor influencing ambulance use. Billittier et al. [20] reported that Medicaid recipients accounted for 59% of unnecessary ambulance transports in New York State (United States). Rucker et al. [12] studied ambulance use among patients who visited the emergency departments of urban teaching hospitals in the northeastern United States and reported that those whose household income was less than $15,000 per year were 1.4 times more likely to use an ambulance than those with a higher income. The present study also showed that household income negatively influences the decision to call an ambulance when a family member faces a nonemergency situation.

User charges were not shown to influence ambulance calls in this study. It is likely that the demands for an ambulance do not decrease consistently with increases in the price [28] and that the price range presented in the study would not alter the demand significantly.

The five socioeconomic factors, i.e., age, living alone, nonpossession of car, nonhesitation, and lack of knowledge of primary emergency medical centers, significantly influenced the hypothetical decisions to call an ambulance in our study.

Elderly persons were shown to be more likely than younger persons to call an ambulance unnecessarily. There are probably several reasons for this trend; for instance, the elderly may depend upon public services more than younger persons do. The elderly might be a target population for policies aimed at ensuring appropriate ambulance use. However, several studies have indicated that it is not easy for elderly persons to assess their conditions. It has been reported that stroke and coronary disease, which are diseases that elderly persons more frequently suffer from than younger persons, are not easily recognized despite the necessity of emergency care [34-36]. It is unclear whether programs aimed at reducing inappropriate ambulance use.

The present study suggested that persons living alone are approximately 5% more likely than those not living alone to call an ambulance in unnecessary situations, and that persons who do not have a car are 2% to 7% more likely to call an ambulance unnecessarily than those with a car. Lacking a means of transport to a hospital or clinic is likely to influence the decision to call an ambulance. Camasso-Richardson et al. [24] reported, from a study of pediatric ambulance transport in the United States, that the main reason for calling an ambulance was lack of another means of transportation and that more than 60% of ambulance transports were considered medically unnecessary. Emergency ambulance crews in the United Kingdom and United States can decide at the scene not to convey patients to emergency departments when patients agree with the crew's assessment that conveyance is not necessary. This policy would reduce costs. However, this non-conveyance policy depends heavily on the crew's ability to make appropriate decisions, and several studies have indicated that paramedics cannot safety determine which patients do not need ambulance transport [37-40]. Although alternative transport services for individuals without a medical emergency deserve to be promoted, the safety of alternative services for emergency transport should be cautiously studied.

The present study suggested that persons who feel no hesitation tend to call an ambulance more easily than those who do feel hesitation; respondents who responded feeling no hesitation were 2% to 5% more apt to state that they would call an ambulance than were respondents who reported feeling hesitation. Such hesitation has been reported as an important factor restricting the decision to call an ambulance even in an emergency situation [28]. Perhaps educational campaigns for appropriate ambulance use have potential to reduce the number of ambulance calls. However, there is neither good evidence that educational campaigns improve the appropriateness of ambulance use nor assurance that educational campaigns can reduce only inappropriate ambulance calls. Further research, such as an intervention study, on the effects of educational campaigns is needed.

Results of the study also suggest that persons who do not know of a primary emergency medical center in the city tend to call an ambulance more easily than those who do know of such a center; respondents who reported not knowing of a primary emergency medical center were 2% to 4% more apt to state that they would call an ambulance than respondents who did know of such a center. Lack of information on primary emergency medical services is likely to influence the decision to call an ambulance. Providing information to the public on the city's primary emergency medical services may have potential to reduce unnecessary ambulance use.

### Limitations

Our study was limited in several ways. First, although there are many situations for which an ambulance call is inappropriate, the hypothetical situations presented in the questionnaire were few. Although the socioeconomic factors that were shown in the study to influence the decision to call an ambulance were nearly the same for all three situations, further research that tests various other situations is needed to clarify the socioeconomic factors that consistently influence the decision to call an ambulance, regardless of the situation. Second, this study used hypothetical scenarios to elicit intention regarding ambulance use rather than data from actual calls. With the dichotomous choice method, there can be a discrepancy between a hypothetical "yes" and a real "yes" [41-43]. Respondents may have been inclined to answer "no" to the questions in which nonemergency situations were presented. Third, the study was conducted in a large urban city. The results of the study may not be applicable to rural areas. Fourth, the questionnaire response rate was 60%; thus, the survey data may not be representative of the whole target population. Non-response bias is always a possibility in a self-administered questionnaire-based survey. In our study, elderly respondents aged 65 years or over accounted for 41.4% of the total respondents. Thus, results of the study may have tended to reflect elderly persons' intentions, although, we adjusted for the influence of age by means of multivariate regression analyses. Another potential bias, that persons who call an ambulance easily might tend not to participate in such a survey, is difficult to resolve.

## Conclusion

Results of the study suggest that several socioeconomic factors, i.e., age, gender, household income, and possession of a car, influence a person's decision to call an ambulance in nonemergency situations. Also, hesitation to use an ambulance and knowledge of the city's primary emergency medical centers are likely to be important factors limiting ambulance overuse. It was estimated that unnecessary ambulance use is increased by approximately 10% to 20% by socioeconomic factors. Education for appropriate ambulance use, promotion of less resource-intensive transportation systems than an ambulance system, and provision of information on the city's primary emergency medical services deserve consideration as policy interventions. However, the possibility of policy interventions and whether they make a notable difference requires further study.

## Competing interests

The author(s) declare that they have no competing interests.

## Authors' contributions

CK and KO designed and managed the study, developed the study hypotheses, and conducted the data analysis. CK drafted the paper. KK and OT contributed to interpreting the data. All authors contributed substantially to the revision of the manuscript.

## Pre-publication history

The pre-publication history for this paper can be accessed here:


